# Successful diagnosis and treatment of autoimmune hepatitis leading to liver failure following PD-1 immunotherapy in a patient with nasopharyngeal carcinoma: a case report

**DOI:** 10.3389/fonc.2026.1806957

**Published:** 2026-04-28

**Authors:** Yu Peng, Yuan Xiao, Yonghu Zhang, Hongyu Jia

**Affiliations:** 1 Department of Infectious Diseases, Beilun District People’s Hospital of Ningbo City (Beilun Branch of the First Affiliated Hospital of Zhejiang University School of Medicine), Ningbo, Zhejiang, China; 2 Department of Neurology, Beilun District People’s Hospital of Ningbo City (Beilun Branch of the First Affiliated Hospital of Zhejiang University School of Medicine), Ningbo, Zhejiang, China; 3 Department of Infectious Diseases, First Affiliated Hospital, Zhejiang University School of Medicine, Hangzhou, Zhejiang, China

**Keywords:** autoimmune hepatitis, case report, immune checkpoint inhibitor, liver failure, nasopharyngeal carcinoma

## Abstract

**Background:**

Immune checkpoint inhibitors (ICIs), particularly programmed cell death protein 1 (PD-1) inhibitors, are widely used in oncology but can cause immune-related adverse events, including hepatotoxicity. Severe ICI-induced liver injury progressing to acute or subacute liver failure carries a poor prognosis and presents a significant management challenge.

**Case presentation:**

We present the case of a 58-year-old male with recurrent nasopharyngeal carcinoma who developed severe autoimmune hepatitis and subacute liver failure following his first cycle of tislelizumab (an anti-PD-1 antibody) combined with chemotherapy. Despite initial management with corticosteroids and hepatoprotective agents, his liver function deteriorated rapidly, characterized by progressive hyperbilirubinemia and coagulopathy. After transfer to a tertiary center, he was managed with high-dose intravenous methylprednisolone, mycophenolate mofetil, and three sessions of artificial liver support (plasma exchange and bilirubin adsorption). His liver function gradually stabilized and ultimately recovered. At follow-up, he achieved normal liver biochemistry and a good quality of life.

**Conclusion:**

This case underscores the potential severity of ICI-induced hepatotoxicity. It highlights the critical importance of early recognition, prompt grading according to guidelines (e.g., CSCO), and aggressive, multimodal immunosuppressive therapy combined with artificial liver support in managing life-threatening immune-mediated liver failure. Close monitoring of liver function before, during, and after ICI therapy is essential.

## Introduction

Nasopharyngeal carcinoma (NPC) is often diagnosed at an advanced stage. While concurrent chemoradiotherapy is the mainstay, immune checkpoint inhibitors (ICIs) have emerged as a promising therapeutic strategy for recurrent or metastatic disease (1). Tislelizumab, a humanized IgG4 monoclonal antibody against PD-1 (2, 3), is approved in China for this indication. By minimizing Fc fragment binding to macrophages, it aims to preserve the number and function of effector T cells, potentially enhancing its anti-tumor activity.

However, ICIs can induce immune-related adverse events (irAEs) across various organs (4). ICI-related hepatotoxicity, though less common than gastrointestinal or dermatologic toxicity, can be severe and life-threatening. The incidence of any-grade ICI-associated hepatotoxicity ranges from 5% to 15%, with grade 3–4 events occurring in 1-4% of patients, depending on the ICI agent and combination regimen. Hepatotoxicity typically occurs within 8–12 weeks of initiation, but can present earlier or later (5).

According to the Chinese Society of Clinical Oncology (CSCO) guidelines, grade 3–4 hepatotoxicity requires prompt high-dose corticosteroid therapy and permanent discontinuation of ICI therapy (6). For steroid-refractory cases, the addition of second-line immunosuppressants such as mycophenolate mofetil is recommended, while the use of infliximab is generally avoided due to the risk of hepatotoxicity. In cases progressing to liver failure, artificial liver support systems (e.g., plasma exchange, bilirubin adsorption) can serve as a bridging therapy to remove inflammatory mediators and toxins, stabilizing the patient while immunosuppressive therapy takes effect (6).

Although tislelizumab is increasingly used in clinical practice, published data on its specific hepatotoxicity profile remain limited. Reports detailing the successful rescue of ICI-induced subacute liver failure with a combination of pharmacologic immunosuppression and extracorporeal liver support are scarce. This case report describes the diagnostic and therapeutic journey of a patient with NPC who developed severe autoimmune hepatitis and liver failure after PD-1 blockade, successfully treated with a protocol of glucocorticoids, mycophenolate mofetil, and serial artificial liver support. We aim to provide a management reference for this severe complication.

## Case presentation

A 58-year-old Chinese male was admitted to the Infectious Diseases Department on Day 56 after his first dose of immunotherapy with chief complaints of fever and jaundice for over 40 days. The patient had no history of alcohol consumption, tobacco use, or drug allergies. He reported no family history of autoimmune diseases or liver disorders. Psychosocial history was unremarkable, with no significant stress or mental health issues identified during the illness course.

On admission, his vital signs were stable: temperature 36.8°C, heart rate 88 beats/min, respiratory rate 18 breaths/min, and blood pressure 128/76 mmHg. Physical examination revealed scleral icterus and generalized jaundice of the skin. Abdominal examination was unremarkable, with no tenderness, hepatosplenomegaly, or ascites. Neurological examination showed normal mental status, no asterixis, and intact motor and sensory function.

His medical history included nasopharyngeal carcinoma (diagnosed >10 years prior), treated with radiotherapy and chemotherapy in 2014 and again in 2019, with normal long-term liver function follow-up after each treatment course. In 2023, upon tumor recurrence, he received his first cycle of immunotherapy with tislelizumab (200mg intravenous) on Day 1, followed by gemcitabine and cisplatin chemotherapy on Day 2. No prior hepatotoxic medications, herbal remedies, or dietary supplements were used.

Approximately two weeks post-immunotherapy(Day 14), he developed a fever (38.6°C) with fatigue and anorexia, but no other specific symptoms. Initial liver function tests were normal. He received moxifloxacin empirically. By Day 19, follow-up tests revealed acute hepatitis (ALT 560 U/L, AST 469 U/L). ICI-induced liver injury was suspected. Low-dose intravenous methylprednisolone (40mg daily) was initiated alongside hepatoprotective agents (e.g., magnesium isoglycyrrhizinate, glutathione).

**Figure 1:**
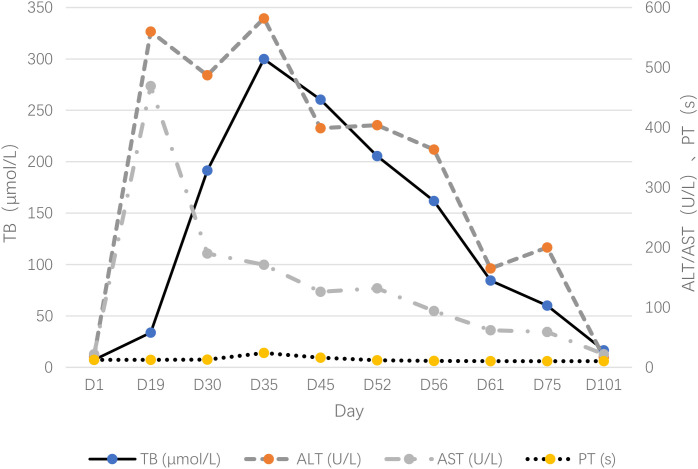
Trend chart of liver function parameters and key therapeutic interventions.

Despite this, the patient developed progressive jaundice and worsening liver biochemistry (**Figure 1**). The steroid dose was escalated stepwise to methylprednisolone 80mg twice daily by Day 30. According to CSCO guidelines, high-dose corticosteroids (1–2 mg/kg) are recommended for grade 3–4 hepatotoxicity; the dose was escalated because liver enzymes and bilirubin continued to rise after 3 days of initial treatment (6). Given ongoing deterioration (Total Bilirubin/TB rising to 299.9 µmol/L by Day 35, with prolonged prothrombin time), mycophenolate mofetil (1g daily) was added on Day 33. Mycophenolate mofetil was selected as the second-line immunosuppressant because it is recommended for steroid-refractory ICI-hepatotoxicity, whereas infliximab is generally avoided due to the potential risk of hepatotoxicity. Prophylactic micafungin was started concurrently. No liver biopsy was performed due to rapidly progressive coagulopathy (PT 24s) and high bleeding risk, which precluded safe histopathological confirmation.

Due to signs of incipient liver failure (hyperbilirubinemia, coagulopathy), he was transferred to a tertiary hospital on Day 39. Treatment with high-dose methylprednisolone (80mg twice daily), mycophenolate mofetil, and broad-spectrum antibiotics was continued. Given the relentless rise in bilirubin, he underwent three sessions of artificial liver support between Day 45 and Day 52, incorporating plasma bilirubin adsorption and dual plasma molecular adsorption system (DPMAS). Artificial liver support was initiated as a bridging strategy to remove inflammatory cytokines, bilirubin, and potential autoantibodies, thereby stabilizing the patient while immunosuppressive therapy took effect (6–10). Following this, his bilirubin levels began a consistent downward trend. The improvement was attributed to the combined effect of intensified immunosuppression and the removal of inflammatory mediators and bilirubin via artificial liver support, rather than either modality alone.

He was transferred back to our hospital on Day 56 for consolidation therapy. Laboratory tests showed TB 226.8 µmol/L, DB 195.6 µmol/L, and normalizing coagulation (PT 11.2s). Methylprednisolone was tapered gradually: reduced from 80mg twice daily to 60mg twice daily on Day 61, and further decreased by 10mg every 5–7 days thereafter based on clinical stability and serial liver function monitoring. Mycophenolate mofetil was continued at 1g daily during the acute phase and was reduced to 0.5g twice daily after discharge, with a planned total duration of at least 3 months before reassessment. He received one additional session of plasma exchange on Day 61, which reduced TB to 84.5 µmol/L. The patient’s clinical condition improved markedly, with resolution of fever and jaundice. He was discharged on Day 75. Outpatient follow-up on Day 101 confirmed complete normalization of liver function tests.

The patient reported progressive improvement in his energy level and overall well-being following stabilization of liver function. At the follow-up visit, he expressed gratitude for the multidisciplinary care and was able to resume normal daily activities. He provided written informed consent for the publication of this case report.

The trend of changes in liver function indicators during the patient’s diagnosis and treatment process is shown in **Figure 1**.A timeline of key therapeutic interventions is summarized in **Table 1**.

**Table 1:** Timeline of key laboratory parameters and therapeutic interventions.

Date	Event	ALT/AST (U/L)	Table (µmol/L)	PT (s)	Intervention
Day 1	ICI initiation	Normal	Normal	Normal	Tislelizumab + chemotherapy
Day 14	Symptom onset	Normal	Normal	Normal	–
Day 19	Hepatitis detected	560/469	–	–	Methylprednisolone 40 mg/d
Day 30	Dose escalation	–	–	–	Methylprednisolone 80 mg bid
Day 33	MMF added	–	–	–	MMF 1 g/d
Day 35	Peak liver injury	–	299.9	Prolonged	–
Day 39	Transfer	–	–	–	–
Day 45–Day 52	Artificial liver support	–	–	–	3 sessions (DPMAS + adsorption)
Day 56	Readmission	–	226.8	11.2	–
Day 61	Plasma exchange	–	–	–	1 session
Day 75	Discharge	–	84.5	–	–
Day 101	Follow-up	Normal	Normal	Normal	–

ALT, alanine aminotransferase; AST, aspartate aminotransferase; TB, total bilirubin; PT, prothrombin time; ICI, immune checkpoint inhibitor; MMF, mycophenolate mofetil; DPMAS, dual plasma molecular adsorption system.

Table is shown as a solid line, ALT/AST as dashed lines, and PT as a dotted line, demonstrating the peak of liver injury followed by gradual recovery after multimodal intervention.

## Discussion

The diagnosis of ICI-induced autoimmune hepatitis (ICI-AIH) is one of exclusion. In this patient, viral hepatitis (A, B, C, E), other systemic infections (EBV, CMV, HIV), autoimmune diseases (anti-nuclear antibody, anti-smooth muscle antibody, etc.), and vascular or biliary causes were rigorously ruled out via serology, imaging, and clinical assessment. The temporal link between tislelizumab initiation and symptom onset (within 3 weeks), coupled with a Roussel Uclaf Causality Assessment Method (RUCAM) score of 10 (“highly probable”) (11), strongly supported the diagnosis of drug-induced liver injury (DILI) with an autoimmune phenotype (12). Although liver biopsy is the gold standard for confirming autoimmune hepatitis, it was contraindicated due to severe coagulopathy (PT 24s) at the time of peak disease activity, representing a practical limitation commonly encountered in fulminant presentations.

Management followed the CSCO guidelines for ICI toxicity (6). For grade 4 hepatotoxicity (AST/ALT>10xULN and/or TB>5xULN), permanent ICI discontinuation and high-dose corticosteroids (1–2 mg/kg methylprednisolone equivalent) are mandated. The lack of response within 3 days warranted the addition of a second-line immunosuppressant; we selected mycophenolate mofetil. An important aspect of this case was the patient’s suboptimal response to high-dose corticosteroids. The mechanisms underlying steroid resistance in ICI-AIH are not fully elucidated but may involve the persistence of overactivated, steroid-insensitive T cells and the continuous production of inflammatory cytokines within the hepatic microenvironment (8). This phenomenon underscores the necessity for early add-on immunosuppressive therapy.

The unique and crucial aspect of this case was the integration of repeated artificial liver support. This intervention likely bridged a critical period by removing inflammatory cytokines, bilirubin, and potential autoantibodies, thereby stabilizing the patient while immunosuppressive therapy took effect (7, 9, 10). Importantly, the observed clinical and biochemical improvement occurred concurrently with both aggressive immunosuppression and extracorporeal support; thus, the favorable outcome likely reflects a synergistic effect rather than the result of any single intervention.

This case has several strengths. It provides a detailed, longitudinal account of a rare but life-threatening complication of PD-1 therapy, with comprehensive documentation of clinical, laboratory, and therapeutic data. The use of a multimodal approach combining guideline-based immunosuppression with artificial liver support offers a practical management template for similar cases. The complete recovery of liver function, confirmed by long-term follow-up, demonstrates the potential for full reversibility even in severe presentations.

However, several limitations should be acknowledged. First, the absence of histopathological confirmation limits definitive characterization of the hepatic injury pattern, though this was unavoidable due to prohibitive bleeding risk. Second, the simultaneous initiation of intensified immunosuppression and artificial liver support precludes precise attribution of the therapeutic effect to either intervention alone. Third, as a single case report, the findings may not be generalizable to all patients with ICI-induced hepatotoxicity, particularly those with different ICI agents or underlying liver diseases.

This case illustrates several key learning points: 1) ICI-AIH can have a fulminant course, progressing to liver failure despite early steroid initiation. 2) A low threshold for escalating therapy (adding mycophenolate mofetil) is crucial in steroid-refractory cases. 3) Artificial liver support is a valuable adjunctive therapy in severe cases with high bilirubin burden and can be lifesaving. 4) Successful management requires a multidisciplinary approach involving oncology, hepatology, and intensive care. 5) Tapering of immunosuppressive therapy should be individualized based on clinical response and biochemical stability to avoid relapse.

Future challenges include identifying predictive biomarkers for severe hepatotoxicity and refining protocols for combining immunosuppressive agents with extracorporeal support.

## Conclusions

This report details the successful management of a life-threatening complication of modern cancer therapy. It demonstrates that severe ICI-induced autoimmune hepatitis progressing to subacute liver failure can be reversed with a timely, aggressive, and multimodal approach combining high-dose corticosteroids, second-line immunosuppression (mycophenolate mofetil), and serial artificial liver support. As the use of ICIs expands, clinicians must maintain a high index of suspicion for hepatotoxicity, monitor liver function vigilantly, and be prepared to escalate management rapidly in severe cases to improve patient outcomes.

## Data Availability

The original contributions presented in the study are included in the article/**Supplementary Material**. Further inquiries can be directed to the corresponding authors.

## References

[1] Guo L Zhou Y Ye Z Li X Chen H Wang Y . Clinical efficacy of tislelizumab combined with induction chemotherapy followed by concurrent chemoradiotherapy in locally advanced nasopharyngeal carcinoma patients. Med Innovation China. (2022) 19:10–4. doi: 10.3969/j.issn.1674-4985.2022.30.003. PMID: 35900448

[2] Wang R Li J Xu XY Liu S Zhao J Sun W . Chinese expert consensus on diagnosis and treatment of immune checkpoint inhibitor-related liver injury (2024 edition). Chin J Hepatol. (2024) 32:121–30. doi: 10.3760/cma.j.cn501113-20240115-00018. PMID: 41912385

[3] Kong L Wang Y Zhang L Hu Q Chen J Yang K . Management of corticosteroid-refractory immune checkpoint inhibitor-related hepatitis: A multicenter retrospective study. Hepatol Int. (2023) 17:890–900. doi: 10.1007/s12072-023-01567-x. PMID: 41933263

[4] Li M Chen X Liu Y Wang H Zhang Q Zhou L . Artificial liver support system as a bridge therapy in immune checkpoint inhibitor-induced acute liver failure: A case series and literature review. Front Immunol. (2022) 13:1001823. doi: 10.3389/fimmu.2022.1001823. PMID: 36119055 PMC9478575

[5] Wang DY Salem JE Cohen JV Chandra S Lutzk A Ghebremichael M . Fatal toxic effects associated with immune checkpoint inhibitors: A systematic review and meta-analysis. JAMA Oncol. (2018) 4:1721–8. doi: 10.1001/jamaoncol.2018.3923. PMID: 30242316 PMC6440712

[6] Chinese Society of Clinical Oncology (CSCO) . Guidelines for toxicity management of immune checkpoint inhibitors 2023. Beijing: People's Health Publishing House (2023).

[7] Martin ED Michot JM Rosmorduc O Saltz JB Lowery MA El Dika I . Liver toxicity as a limiting factor to the increasing use of immune checkpoint inhibitors. JHEP Rep. (2020) 2:100170. doi: 10.1016/j.jhepr.2020.100170. PMID: 33205034 PMC7648167

[8] Xing H Wang Y Qu B Li J Zhang Y Liu X . The current status of steroid-refractory immune-checkpoint-inhibitor-related hepatotoxicity. Transl Oncol. (2023) 28:101619. doi: 10.1016/j.tranon.2023.101619. PMID: 36623392 PMC9842701

[9] Moi L Bouchaab H Mederos N Kleiner DE Abou-Alfa GK Chan J . Personalized cytokine-directed therapy with tocilizumab for refractory immune checkpoint inhibitor-related cholangiohepatitis. J Thorac Oncol. (2021) 16:318–26. doi: 10.1016/j.jtho.2020.09.009. PMID: 32956849

[10] Bai L Chen Y Severe Liver Disease and Artificial Liver Group Hepatology Branch Chinese Medical Association . Expert consensus on clinical application of artificial liver blood purification technology (2022 edition). J Pract Hepatol. (2022) 25:457–68. doi: 10.3969/j.issn.1672-5069.2022.03.039. PMID: 35900448

[11] Professional Committee for Prevention and Treatment of Drug-induced Liver Injury of China Medical Biotechnology Association Drug-induced Hepatology Group Hepatology Branch Chinese Medical Association . Guidelines for diagnosis and treatment of drug-induced liver injury in China (2023 edition). Chin J Hepatol. (2023) 31:355–84. doi: 10.3760/cma.j.cn501113-20230419-00176. PMID: 37248972 PMC12854814

[12] Björnsson E Talwalkar J Treeprasertsuk S Burns A Lindor K . Drug-induced autoimmune hepatitis: clinical characteristics and prognosis. Hepatology. (2010) 51:2040–8. doi: 10.1002/hep.23588. PMID: 20512992

